# Psychometric Properties of the Turkish Version of the Children’s Saving Inventory in a Clinical Sample

**DOI:** 10.5152/eurasianjmed.2023.23102

**Published:** 2023-10-01

**Authors:** Mehmet Akif Akıncı, Bahadır Turan, Ali Çakır, İbrahim Selçuk Esin, Eric Alan Storch, Onur Burak Dursun

**Affiliations:** 1 Department of Child and Adolescent Psychiatry, Atatürk University Faculty of Medicine, Erzurum, Turkey; 2 Department of Child and Adolescent Psychiatry, Karadeniz Technical University Faculty of Medicine, Trabzon, Turkey; 3 Department of Child and Adolescent Psychiatry, Regional Training and Research Hospital, Erzurum, Turkey; 4 Department of Child and Adolescent Psychiatry, Health Science University Faculty of Medicine, Trabzon, Turkey; 5 Department of Psychiatry and Behavioral Sciences, Baylor College of Medicine, Houston, USA; 6 Department of Turkish Ministry of Health, General Directorate of Health Services, Ankara, Turkey

**Keywords:** Hoarding, obsessive-compulsive disorder, Children’s Saving Inventory, reliability, validity

## Abstract

**Objective:**

The Children’s Saving Inventory (CSI) is a measurement tool developed to assess hoarding behavior in children. This study aims to investigate the psychometric properties of the Turkish version of the CSI in a clinical sample of children and adolescents.

**Materials and Methods:**

The study sample consisted of 52 children and adolescents diagnosed with obsessive-compulsive disorder in the 8-17 age group and their families. As a structured diagnostic interview, the Development and Well-Being Assessment (DAWBA) was applied to all participants included in the research. Hoarding disorder (HD) diagnosis was made clinically by considering the Diagnostic and Statistical Manual of Mental Disorders, Fifth Edition (DSM-5) diagnostic criteria. The Children’s Yale-Brown Obsessive–Compulsive Scale Symptom Checklist (CY-BOCS) was administered by an experienced clinician. The parents and children filled out the Obsessive-Compulsive Inventory—Child Version (OCI-CV) and CSI scales independently.

**Results:**

The 20-item CSI Turkish version demonstrated good internal consistency. This 4-factor structure of the scale was confirmed by confirmatory factor analysis. Children’s Saving Inventory showed convergent and discriminant validity with the OCI-CV and CY-BOCS subscales, and the higher CSI total scores in children and adolescents diagnosed with HD confirmed the construct validity.

**Conclusion:**

These findings support the use of the CSI Turkish version as a valid and reliable scale to investigate the hoarding behavior of children and adolescents in a clinical sample. In addition, the CSI Turkish version is currently the only validated instrument to evaluate hoarding behavior in children and adolescents, as rated by parents in Türkiye.

**Main Points**

- This is the first and only study to test the validity and reliability of the Children’s Saving Inventory (CSI) in another culture.
- The CSI Turkish version demonstrated good internal consistency.
- This four-factor structure of the scale was confirmed by confirmatory factor analysis.
- The CSI Turkish version was found to be a valid and reliable scale for use in a clinical sample.

## Introduction

Hoarding disorder (HD) is a condition where individuals experience difficulties getting rid of possessions and have strong attachments to them, leading to distress when faced with the prospect of discarding them.^1^ While hoarding was previously considered a symptom of obsessive-compulsive disorder (OCD), HD is now recognized as a separate disorder within the OCD and Related Disorders category in the Diagnostic and Statistical Manual of Mental Disorders, Fifth Edition (DSM-5).^2^ Research indicates that HD typically emerges between the ages of 11 and 15,^3^ although there is limited data available for childhood and adolescence in the scientific literature. However, the number of studies on this age group has been increasing since the inclusion of HD in the DSM-5. The estimated prevalence of HD in children and adolescents is 0.98%.^4^

It is emphasized that the early diagnosis and treatment of HD is important in terms of preventing the future recurrence of problems as well as increasing functionality in adult life.^5^ Evidence-based assessments encourage the routine use of standard tools for the screening, diagnosis, and follow-up of psychiatric disorders in young people.^6^ Self-report questionnaires are widely used as an important source of information in daily practice since they help children and adolescents report their feelings, thoughts, and behaviors. Because hoarding is a subset of OCD, hoarding behavior is assessed using 2 items related to hoarding obsessions/compulsions on the Children’s Yale-Brown Obsessive–Compulsive Scale Symptom Checklist (CY-BOCS)^7^ and several items on the Obsessive-Compulsive Inventory—Child Version (OCI-CV).^8^ Hoarding disorder being recognized as a separate disorder and the limited ability of OCD scales to measure hoarding behaviors have led to the development of a specific scale for measuring hoarding in children and adolescents. Storch et al^9^ (2011) created the Children’s Saving Inventory (CSI), which is a parent-rated measure of a child’s level of hoarding behavior. The CSI is the first and only tool designed to assess hoarding behavior in children.

The CSI is based on the Saving Inventory-Revised (SI-R), a well-established adult self-report scale with strong psychometric properties.^10^ The items of the SI-R were revised to be appropriate for children and to be completed by parents. The psychometric properties of the CSI were tested on American^9^ and Canadian^11^ children and adolescents diagnosed with OCD. While the scale developers assessed the psychometric characteristics of the original 4-factor 23-item scale in the American context, the Canadian team assessed the revised 3-factor 15-item version, which excluded the factor assessing clutter. However, no validity or reliability study has yet been conducted for this tool in any language other than English. The lack of valid and reliable tools in non-English-speaking countries limits the ability to screen, diagnose, treat, and follow up on psychiatric disorders. Regardless of being shown to be sufficient in 1 culture, it is not guaranteed that these diagnostic instruments will be valid or reliable in another culture.^12^ There are differences between different cultures in the demographic characteristics, severity of symptoms, and comorbid psychiatric disorders of hoarding.^13^ Therefore, it is critical to translate the CSI parent version into a language other than English and to test it in a non-English-speaking culture.

This study aims to investigate the psychometric characteristics of the Turkish CSI rated by parents in a clinical sample of children and adolescents in the 8-17 age group. We hypothesized that the results of the study would support the validity and reliability of the original 4-factor CSI in Turkish society.

## Materials and Methods

### Procedure and Participants

Necessary written permission and ethical approval for the study were obtained from the Ethics Committee of Atatürk University (B.30.2.0.01.00/50-01/29). In addition, permission was obtained electronically from the authors of the scale. After obtaining the necessary permissions, the items in the CSI were translated into Turkish separately by the present authors (fluent English speakers); then, the differences in the translated questionnaire were checked at a meeting. The inconsistencies were then examined by another researcher (a native English speaker) who was blind to the original items. The final version was accepted by all team members. The Turkish translation was translated back into English by a clinician who was fluent in both languages, and the translation was submitted to the author, who granted permission to use the original CSI. The participants included 52 children and adolescents aged 8-17 and their families. The children and adolescents were admitted to our outpatient clinic, where they were diagnosed with OCD for the first time. Those who read the informed consent form, volunteered to participate in the research, and signed the written consent form were included in the study. As a structured diagnostic interview, the Development and Well-Being Assessment (DAWBA) was applied to all participants included in the research. Obsessive-compulsive disorder diagnoses and comorbid psychiatric diagnoses were made using the DAWBA. Children and adolescents with a primary diagnosis of OCD were included in the study. The primary diagnosis was determined by the clinicians based on psychiatric interviews and the tools used according to the clinical manifestation that caused the most distress and deterioration in functionality. In addition, clinical interviews were conducted to assess the DSM-5 diagnostic criteria. The DSM-5 diagnostic criteria were endorsed. The findings and materials obtained as a result of the psychiatric interview were reviewed by an experienced child and adolescent psychiatrist. Each criterion and specifier of HD has been endorsed. Whether it is appropriate to use the C diagnostic criterion in children and adolescents and whether it should be adapted due to the strictness of this criterion remain controversial issues. Therefore, we neither waived this criterion nor disregarded parental intervention when confirming criterion C. Hence, criterion C was validated based on the verbal statement of the parent. At the end of all procedures, HD in children and adolescents was diagnosed based on the DSM-5 diagnostic criteria. Participants who had received or were already receiving treatment were excluded from the research due to their potential effects on the diagnosis and clinical presentation of both HD and OCD. The CY-BOCS was applied by an experienced clinician. The parents, children, and adolescents completed the OCI-CV and CSI scales independently.

### Materials

#### Sociodemographic Data Form

The form prepared within the scope of the study was used to collect demographic information (e.g., age, gender, grade, duration of maternal and paternal education, income status, etc.) about the children and their parents.

#### Development and Well-Being Assessment

This is a diagnostic tool used to assess psychiatric disorders in children and adolescents aged 2-17 years. It uses both the 10th edition of the International Classification of Diseases (ICD-10) and the DSM IV-V classifications. The DAWBA is composed of 3 parts: a structured interview for parents, a structured interview for young people aged 11-17, and a questionnaire for teachers. The interviews can be conducted through a written interview text or a computer application and can also be completed by parents, young people, and teachers themselves without the need for an interviewer. Unlike other interviewer-based formats, DAWBA includes open-ended questions in each section. This allows for a more accurate evaluation of symptoms and loss of functionality.^14^ The Turkish version of DAWBA was developed by Dursun et al^15^ in 2013.

#### Children’s Saving Inventory

The CSI is a parent-rated tool developed by Storch et al^9^ to measure the frequency and severity of hoarding symptoms in children aged 8-17 years with OCD. The scale ranges from 0 to 4, with higher scores indicating more severe hoarding symptoms. The original scale consisted of 23 items, which were tested on 123 children and adolescents with OCD and their parents. Three items were removed from the scale due to low correlations and factor loadings. The final 23-item scale was found to be valid and reliable in the United States, with an internal consistency coefficient of *r* = .84-.96. In this study, the original 23-item version of the scale was used to assess the suitability of the scale for use in the Turkish population.

#### Children’s Yale-Brown Obsessive—Compulsive Scale

This is a semi-structured interview form used to assess the reported severity of obsessive-compulsive symptoms.^7^ Obsessions and compulsions are scored in 5 subscales (scored from 0 to 4 points per item) to calculate the CY-BOCS obsession score (0-20 points), the CY-BOCS compulsion score (0-20 points), and the CY-BOCS total score (0-40 points). The Turkish reliability study of the scale was conducted in 2006 by Yücelen et al.^16^

#### Obsessive-Compulsive Inventory—Child Version

This is a self-reported Likert-type instrument used to measure the level of obsessive-compulsive symptoms in children and adolescents.^8^ The scale consists of 6 subscales: doubt/checking, obsessions, neutralizing, washing, ordering, and hoarding. Each item of the 21-item scale is scored from 0 to 4 points, with increasing scores in each subscale indicating more prominent OCD symptoms. The Turkish validity and reliability study of the scale was carried out in 2014 by Seçer.^17^

### Statistical Analysis

The Statistical Package for the Social Sciences version 24.0 (IBM SPSS Corp.; Armonk, NY, USA) program was used to analyze the data obtained in the study. The Kolmogorov–Smirnov test was used to test the normality of the distribution of continuous variables. An independent sample *t*-test was used for between-gender and between-group (HD/non-HD) comparisons. Continuous variables were presented using means and standard deviations (mean ± SD), and the categorical variables were presented as numbers and percentages (n, %).

The internal consistency of the CSI total and subscale scores was evaluated by examining Cronbach’s alpha coefficients, item-total correlations, and Cronbach’s alpha if the item was deleted. For acceptable reliability, alpha values should be in the range of 0.70-0.95.^18,19^ One method to assess whether the items need to be removed from a scale to improve its alpha coefficient is to calculate the adjusted item-total correlation and remove items with a low (≤0.30) correlation.^19,20^ To achieve adequate item-total correlation values for the Turkish CSI scale, we used a cut-off value of 0.30, which is the accepted general cut-off value for the item removal criterion.

Confirmatory factor analysis (CFA) was performed using AMOS 24.0 software to test the model fitness of the original 23-item CSI and the 20-item Turkish CSI, which were developed by removing items that did not meet the criteria. To assess fitness, various fit indices were investigated, including model *χ* ^2^, *df*, and *P* values, the root mean square error (RMSEA) of approximation (0.05-0.08 = indicate adequate fitness, values >.05 = good fitness),^21^ the Comparative Fit Index (CFI, 0.90-0.95 = adequate fitness, ≥0.95 = good fitness), and Tucker–Lewis Index (TLI, 0.90-0.95 = adequate fitness, ≥0.95 = good fitness).^22^ In addition to examining model fit indices to obtain the most appropriate model, item factor loadings were also examined to identify items with poor fitness. A threshold value of 0.40 was used to include items on the scale.^23^ As a result, items with low factor loadings and low item-total correlations were removed.

The validity of the Turkish version of the CSI was evaluated using Pearson’s correlation analysis for continuous variables. The correlations between the CSI total and its subscales (discarding, clutter, acquisition, and distress) and between the CSI total, CY-BOCS (obsession, compulsion, and total score), and OCI-CV (doubt/checking, obsessions, neutralizing, washing, ordering, hoarding, and total score) were examined. In addition, the differences between the CSI total and subscale scores of the participants with and without HD diagnoses in the clinical interview were compared to test the construct validity of the CSI. A significance level of *P* < .05 was used to indicate statistical significance.

## Results

### Sociodemographic and Clinical Findings

The sociodemographic characteristics and psychiatric comorbidities of the participants are summarized in Table 1. The sample for this study included 52 children and adolescents with OCD diagnoses (age range: 8-17; average age: 13.67 ± 2.34). Of the participants, 28 (53.8%) were male. The median school year of the children and adolescents was eighth grade (range: 3rd to 12th grade). Comorbid psychiatric disorders were present in 50% (n = 26) of the participants. The most common comorbid psychiatric disorder was found to be attention deficit hyperactivity disorder (21.1%, n = 11). This is followed by generalized anxiety disorder (15.4%, n = 8) and depressive disorder (11.5%, n = 6). There was no statistically significant difference between the genders in terms of the CSI total and subscale (discarding, clutter, acquisition, and distress) scores, the CY-BOCS (obsession, compulsion, and total score), and the OCI-CV (doubt/checking, obsessions, neutralizing, washing, ordering, hoarding, and total score) scores (*P* > .05). In addition, there was no significant correlation between the ages of children and adolescents and CSI total and subscale scores (*P* > .05).

### Reliability

The Cronbach’s alpha reliability of the 23-item original CSI was 0.91. Three items were removed from the scale: Item 2, *How much control does your child have over his/her urges to acquire possessions that s/he does not need?;* Item 3, *How much time do you spend dealing with your child’s possessions (e.g., organizing, discarding, arranging)?*; and Item 4, *How much control does your child have over his/her urges to save possessions that s/he does not need?* The item-total correlations for Items 2, 3, and 4 were lower than 0.30 (0.12, 0.21, and 0.10, respectively); they also showed low factor loadings in CFA (see CFA). After removing these items, the analysis was repeated. The internal consistency of the Turkish 20-item CSI was excellent for the total score (Cronbach’s *α* = 0.93) and the subscales of discarding (*α* = 0.89), clutter (*α* = 0.78), acquisition (*α* = 0.83), and distress/impairment (*α* = 0.80). Item-total correlations ranged from 0.44 to 0.78 for the total scale, 0.57-0.85 for the discarding subscale, 0.43-0.76 for the clutter subscale, 0.43-0.74 for the acquisition subscale, and 0.51-0.73 for the distress/impairment subscale.

### Confirmatory Factor Analysis

The model fit of the original 23-item CSI data was somewhat good (RMSEA = 0.077, TLI = 0.87, and CFI = 0.89). All factor loadings, except for 3 items, were positive and significant (*P* < .001). The factor loads of Item 2 (regression weight = 0.05), Item 3 (regression weight = 0.27), and Item 4 (regression weight = 0.14) were not significant (*P* = .735, *P* = .094, and *P* = .315, respectively). These 3 items were removed from the scale due to low item-total correlations in the reliability analysis and low factor loads in the CFA. Once removed, the analysis was repeated. The 20-item Turkish CSI was found to have an acceptable goodness of fit for the model (RMSEA = 0.072, TLI = 0.0.91, CFI = 0.92). The chi-square test results and fit statistics are shown in Table 2.

### Face Validity

Descriptive statistics and correlations for the CSI total and subscale scores are presented in Table 3. All CSI scores (total, discarding, clutter, acquisition, and distress/impairment) had moderate to high correlations with each other (all *P* < .001).

### Convergent Validity

Correlations between CSI scores and the hoarding subscale scores on the other scales used suggest acceptable convergent validity. The CSI total score was strongly correlated with the hoarding subscale of the OCI-CV (*r* = 0.648, *P* < .001).

### Discriminant Validity

Low and moderate correlations were found between the CSI total score and the CY-BOCS total score (*r* = 0.287, *P* < .05), and between the CSI total score and the OCI-CV total score (*r* = 0.486, *P* < .001). In addition, there was no significant relationship between the CSI total score and the CY-BOCS and OCI-CV subscales (CY-BOCS: obsession and compulsion; OCI-CV: doubt/checking, obsessions, washing, ordering, and neutralizing, *P* > .05). The correlations between the CSI total score and the OCD scales are shown in Table 4.

### Construct Validity

To test construct validity, the CSI total and subscale scores of the participants with (n = 7) and without (n = 45) HD diagnoses, according to clinical interviews, were compared. A statistically significant difference was found between the groups with and without HD diagnosis in CSI total scores (46.43 vs. 20.89, *P* < .001). A statistically significant difference was also found between the groups with and without HD diagnosis in terms of CSI discarding (17.29 vs. 6.22), CSI clutter (7.29 vs. 2.16), CSI acquisition (11.71 vs. 6.33), and CSI distress/impairment (10.14 vs. 6.18) subscales (*P* < .05).

## Discussion

To the best of our knowledge, this is the first and only study to test the validity and reliability of the CSI—the first and only measurement tool developed to evaluate hoarding symptoms in children—in another culture. In general, the Turkish version of the CSI was found to be a valid and reliable scale for use in children and adolescents in a clinical sample.

The scale developers excluded 2 items (Item 2 and Item 4) from the original 23-item CSI from their analysis due to their low item-total correlation and 1 item (Item 11) due to insufficient factor loading in the explanatory factor analysis.^9^ In the analysis performed in our study, Item 2 (*How much control does your child have over his/her urges to acquire possessions that s/he does not need?*) and Item 4 (*How much control does your child have over his/her urges to save possessions that s/he does not need?*) were removed from the scale due to low item-total correlations and low factor loads in CFA. However, Item 11 (*To what extent does attachment to things interfere with your child’s functioning at school, at home, or with friends?*), which did not apply to the US population, was left on the scale since it had good fitness in the Turkish population. On the contrary, Item 3 (*How much time do you spend dealing with your child’s possessions (e.g., organizing, discarding, arranging)?*), which applies to the US population, was removed from the scale due to insufficient fitness in the Turkish population. Thus, the 20-item Turkish CSI was obtained. The 4-factor Turkish CSI-20 was found to have an acceptable goodness of fit for the model. This result confirmed the 4-factor structure of the Turkish population. Compared to the original 23-item CSI, although we do not argue that the original CSI does not fit, the Turkish CSI-20 fits Turkish society better. When these findings are considered together, they offer a good example of how scale items translated into other languages and tested in different cultures may not have similar fitness.

Determining the internal consistency of the scale is one of the most important steps in determining whether a scale is reliable by evaluating item-total correlations.^24^ One method of measuring a scale’s internal consistency is the calculation of Cronbach’s alpha coefficient.^18^ The Cronbach’s alpha value in the study was found to be 0.93. Acceptable and good internal consistency was found for each of the 4 factors (discarding [*α* = 0.89], clutter [*α* = 0.78], acquisition [*α* = 0.83], and distress/impairment [*α* = 0.80]). Moreover, it was observed that all the scale items had positive correlations among themselves and with the total score of the scale. This shows that each item on the scale contributes to the total score. Our findings show that the 20-item CSI Turkish version has excellent reliability. In addition, the calculated Cronbach’s alpha values appear to be close to the values of the original scale (total score [*α* = 0.96], discarding [*α* = 0.95], clutter [*α* = 0.90], acquisition [*α* = 0.94], and distress/Impairment [*α* = 0.84]).^9^

In addition to the reliability analyses, validity analyses show that the Turkish CSI-20 has adequate validity. First, the mutual correlations of the CSI total and subscale scores were examined for face validity. All CSI scores (total, discarding, clutter, acquisition, and distress/impairment) had moderate to high correlations with each other. Second, the strong correlation between the CSI total score and the OCI-CV hoarding subscale supports acceptable convergent validity. Third, low and medium correlations were found between the CSI total score and the CY-BOCS and OCI-CV total scores. Since the sample of the study included children with OCD, this finding is expected. No significant correlations were found between the CSI total score and the other CY-BOCS subscales (obsession and compulsion) and the OCI-CV subscales (doubt/checking, obsessions, washing, ordering, and neutralizing). This, in turn, indicates the discriminant validity of the CSI-20. Finally, to test construct validity, the CSI total and subscale scores of participants with and without HD diagnoses were compared. In this study, HD diagnostic evaluation was based on a combined evaluation of data collected through clinical interviews with both the children and their parents. A statistically significant difference in the CSI total and 4 subscale scores (discarding, clutter, acquisition, and distress/impairment) was found between the groups with and without an HD diagnosis. This result provides evidence that CSI can be used to distinguish between those with and without hoarding behavior.

This study has some limitations despite its strengths. One of the limitations is that, similar to the original CSI, the generalizability of the results may be restricted because the study only focused on children and adolescents with OCD. Therefore, testing the applicability of community sampling and a clinical sample of young people diagnosed with HD would be useful for future studies. Second, the relatively small sample size (n = 52) might be considered a limitation. Third, since the CSI is a parent-rated scale, there is a need for a child version to compare parent–child self-reports. It is unclear how effective the child version of the questionnaire would be for this age group because children and adolescents have limited control and resources in their home environment, and parental involvement in their living spaces could impact the nature, features, and consequences of hoarding behavior. These potential effects will be explored in future studies. Fourth, the authors did not conduct test-retest analyses by repeating questionnaire administration a few weeks after filling out the first questionnaires, which might also be listed among the limitations of this study.

In conclusion, this study investigated the psychometric properties of the Turkish version of a parent-rated scale for the hoarding behavior of children and adolescents in a clinical sample. The 20-item CSI Turkish version demonstrated good internal consistency for both the total score and the factor scores. This 4-factor structure of the scale was confirmed by CFA. Children’s Saving Inventory showed convergent and discriminant validity with the OCI-CV and CY-BOCS subscales, and the higher CSI total scores in children and adolescents diagnosed with HD confirmed the construct validity. As a result, our findings support the idea that the Turkish version of the CSI can be used as a valid and reliable measurement tool to assess the hoarding symptoms of children and adolescents in a clinical sample. We also believe that this study will lead to further validity and reliability studies of the CSI in cultures and languages other than English.

## Figures and Tables

**Table 1.** Sociodemographic Characteristics of Participants (n = 52)

Age (years)	
Mean ± SD; range	13.67 ± 2.34; 8-17 years
Gender, n (%)	
Male	28 (53.8)
Female	24 (46.2)
Grade Median; range	8; 3rd-12th grades
Mothers’ schooling (years)	
Mean ± SD	7.27 ± 4.15
Fathers’ schooling (years)	
Mean ± SD	10.40 ± 3.83
Family income, n (%)	
Not regular income	2 (3.8)
Low	10 (19.2)
Middle	29 (55.8)
High	11 (21.2)
CY-BOCS	
Mean ± SD	24.58 ± 4.31
OCI-CV	
Mean ± SD	60.63 ± 15.28
DAWBA diagnosis, n (%)	
Attention-deficit/hyperactivity disorder	11 (21.1)
Generalized anxiety disorder	8 (15.4)
Depressive disorder	6 (11.5)
Specific phobia	4 (7.7)
Social phobia	3 (5.8)
Oppositional defiant	3 (5.8)
Tic disorder	1 (1.9)

CY-BOCS, Children’s Yale-Brown Obsessive–Compulsive Scale Symptom Checklist; DAWBA, Development and Well-Being Assessment; OCI-CV, Obsessive-Compulsive Inventory—Child Version; SD, standard deviations.

**Table 2.** Fit Statistics for the Confirmatory Factor Analytic Models

Model	*χ* ^2^	*df*	*P*	*χ* ^2^/*df*	RMSEA	CFI	TLI
CSI-original 23 item	334.401	224	<.001	1.492	0.077	0.89	0.87
CSI-TR 20 item	251.111	164	<.001	1.531	0.072	0.92	0.91

*χ* ^2^, Chi square; CFI, Comparative Fit Index; CSI, Children’s Saving Inventory; CSI-TR, Children’s Saving Inventory-Turkish version; *df*, degrees of freedom; RMSEA, root mean square error of approximation; TLI, Tucker–Lewis Index.

**Table 3.** Children’s Saving Inventory Total and Subscale Scores Correlations and Descriptive Statistics

	CSI Total	CSI Discarding	CSI Clutter	CSI Acquisition	CSI Distress
CSI total	1				
CSI discarding	.893**	1			
CSI clutter	.802**	.792**	1		
CSI acquisition	.780**	.495**	.422**	1	
CSI distress	.839**	.624**	.499**	.677**	1
Mean ± SD	24.33 ± 15.29	7.71 ± 5.99	2.85 ± 3.44	7.06 ± 4.64	6.71 ± 4.23
Cronbach’s *α*	0.93	0.89	0.78	0.83	0.80

CSI, Children’s Saving Inventory; SD, standard deviations.

**P < .001.

**Table 4.** Correlations Between CSI Total Score and OCD Scales

	CSI Total
CY-BOCS (obsessions)	.275
CY-BOCS (compulsions)	.193
CY-BOCS (total)	.287*
OCI-CV (checking)	.209
OCI-CV (obsessing)	.260
OCI-CV (hoarding)	.648**
OCI-CV (washing)	.027
OCI-CV (ordering)	.050
OCI-CV (neutralizing)	.275
OCI-CV (total)	.486**

CSI, Children’s Saving Inventory; CY-BOCS, Children’s Yale-Brown Obsessive–Compulsive Scale Symptom Checklist; OCD, obsessive-compulsive disorder; OCI-CV, Obsessive-Compulsive Inventory—Child Version.

*P < .05, **P < .01.
